# Predicting rapid decline in kidney function among type 2 diabetes patients: A machine learning approach

**DOI:** 10.1016/j.heliyon.2024.e40566

**Published:** 2024-11-22

**Authors:** Eri Nakahara, Kayo Waki, Hisashi Kurasawa, Imari Mimura, Tomohisa Seki, Akinori Fujino, Nagisa Shiomi, Masaomi Nangaku, Kazuhiko Ohe

**Affiliations:** a Nippon Telegraph and Telephone Corporation, Japan; b The University of Tokyo, Japan

**Keywords:** Artificial intelligence, Diabetic kidney disease, Machine learning, Rapid decline, Recursive feature elimination

## Abstract

**Background:**

Diabetic kidney disease (DKD) is one of the typical complications of type 2 diabetes (T2D), with approximately 10 % of DKD patients experiencing a Rapid decline (RD) in kidney function. RD leads to an increased risk of poor outcomes such as the need for dialysis. Albuminuria is a known kidney damage biomarker for DKD, yet RD cases do not always show changes in albuminuria, and the exact mechanism of RD remains unclear. Previous studies focused on a limited number of laboratory tests, no comprehensive study targeting a wide range of laboratory tests has been done. We target to develop a model that predicts RD among T2D and points to key laboratory tests of interest in understanding RD from various laboratory tests.

**Methods:**

Our machine learning model predicts whether RD, as represented via eGFR, will happen within 1 year. Additionally, the model uses Recursive feature elimination with cross-validation (RFECV) to eliminate the features that do not contribute to the prediction. We trained and assessed the model using 1202 types of laboratory tests from 3438 diabetes patients at the University of Tokyo Hospital.

**Result:**

The means (95 % confidence interval) of the receiver operating characteristic area under the curve (ROC-AUC), precision-recall area under the curve, accuracy rate, and F1-score of an 8-feature-model were 0.820 (0.811, 0.829), 0.430 (0.410, 0.451), 0.754 (0.747, 0.761), and 0.500 (0.485, 0.515), respectively. The RFECV revealed that 7 test types (MCH, γ-GTP, Cre, HbA1c, HDL-C, eGFR, and Hct) contributed to RD prediction. The model's ROC-AUC of 0.820 improves on the ROC-AUC of 0.775 seen in previous studies.

**Conclusion:**

The proposed model accurately predicts RD among diabetes patients and helps physicians focus on inhibiting progression of kidney damage. The contributing laboratory tests may serve as alternative biomarkers for DKD.

## 1 Introduction

Diabetic kidney disease (DKD) is one of the typical complications of type 2 diabetes (T2D) and affects approximately 20 % of the 400 million individuals with diabetes globally [1]. DKD typically initially presents as moderately increased albuminuria and progresses to severely increased albuminuria as the disease advances. Ultimately, DKD leads to a decline in kidney function, necessitating long-term dialysis therapy. Physicians need to identify early signs of DKD in diabetes patients to support early referral to a nephrologist in order to prepare for dialysis.

In recent years, a subset of diabetes patients has exhibited a sudden and precipitous decline in kidney function, termed Rapid decline (RD) [[2], [3], [4], [5], [6]]. Albuminuria is known to be a kidney damage biomarker in DKD and is commonly seen as diabetes progresses, but RD has a rapid deterioration in kidney function not always associated with prominent albuminuria.

In previous studies, RD has been indicated by the decline in the slope of the estimated glomerular filtration rate (eGFR) greater than 5 mL/min/1.73 m^2^/year [5] or by a loss of eGFR of from 2.8 % to 10 % per year or greater [[2], [7], [8], [9], [10], [11], [12], [13], [21]]. A cohort study in Japan revealed the prevalence of DKD was 52 %, and 12 % of these DKD patients had RD indicated [12]. RD has been reported to be a predictor of future kidney failure [[14], [15], [16]]. Currently, there is little understanding about the mechanisms underlying the progression of RD.

Since some cases of RD do not exhibit changes in albuminuria, studies have worked on detecting signs of RD using alternative methods. One approach is to find biomarkers related to RD, such as the measurement of serum TNF receptors (TNFR) [17] or microRNA [18,19]. However, measuring these items in all diabetes patients within the routine clinical practice of diabetes care is challenging. Many laboratory tests are already being carried out in daily diabetes care, and it would be very useful if signs of RD could be found among them.

Factors associated with RD have been identified and include high hemoglobin A1c (HbA1c), high systolic blood pressure, older age, and low low-density lipoprotein cholesterol (LDL-C). Machine learning (ML) has been used to predict RD using these factors [20,21] (Table 1). A study predicted RD among 19,894 diabetes patients based on changes in eGFR by using the Random Forest method [22], resulting in an ROC-AUC of 0.73 [20]. A second predicted RD with an ROC-AUC of 0.775 using an ML model that uses binary (e.g., sex, family history of diabetes), multi-categorical (e.g., urine nitrous acid (U-NIT), urine urobilinogen (U-UBG)), and quantitative features (e.g., body height and hemoglobin) as inputs [21]. Most studies on predicting RD have focused on a limited number of specific laboratory tests. No comprehensive study targeting a wide range of laboratory tests has been done. When increasing the number of laboratory tests targeted by ML, two challenges are overfitting and missing data [27,28]. This study used Light Gradient Boosting Machine (LightGBM) [29] and Recursive Feature Elimination with Cross-Validation (RFECV) [30] to address these problems. LightGBM is a gradient boosting framework that uses tree-based learning algorithms and is acknowledged for its superior predictive performance [31,32]. Even when given a huge variety of interpolated and quantized laboratory test values as input, LightGBM avoids overfitting with built-in regularization techniques. RFECV enhances accuracy, reduces overfitting, and improves interpretability by iteratively removing less important features to focus on key data.

**Table 1:** Comparison of previous studies using static analysis and ML.

Type	Author	Definition of RD	Target patients	Association factor of RD	Performance index (ROC-AUC)
static analysis	Krolewski et al. (2014)[8]	eGFR decline ≥3.3 %/year	T1D and albuminuria (N = 277)	high HbA1c, high systolic blood pressure, older age, high uric acid value, high TNFR-1, high TNFR-2, low eGFR	–
static analysis	Zoppini et al. (2012) [4]	eGFR decline ≥4 %/year	T2D and albuminuria (N = 699)	high albuminuria, high eGFR, high body mass index, high systolic blood pressure, cardiovascular disease, older age, insulin therapy	–
static analysis	Jiang et al. (2019) [13]	eGFR decline ≥3.1 %/year	T2D and albuminuria (N = 4685)	high HbA1c, low LDL-C, low eGFR, high blood pressure, retinopathy, moderately increased albuminuria	–
static analysis	Vistisen et al. (2019) [9]	eGFR decline ≥2.8 %/year	T2D and albuminuria (N = 857)	no statin, no renin-angiotensin system blockers, older age, low eGFR, less exercise	–
static analysis	Bjornstad et al. (2015) [23]	eGFR decline >3 mL/min/1.73 m^2^/year	T1D (N = 519)	HbA1c, systolic blood pressure, LDL-C, diabetes duration, estimated insulin sensitivity, baseline albumin/creatinine ratio	–
static analysis	Sheen et al. (2014) [24]	eGFR decline >5 mL/min/1.73 m^2^/year	T2D, review paper	ethnic/genetic and demographic causes, smoking habits, increased glycated hemoglobin levels, obesity, albuminuria, anemia, low serum magnesium levels, high serum phosphate levels, vitamin D deficiency, elevated systolic blood pressure, pulse pressure, brachial-ankle pulse wave velocity values, retinopathy, cardiac autonomic neuropathy	–
static analysis	Yokoyama et al. (2011) [10]	eGFR decline ≥4 %/year	T2D (N = 1100)	high eGFR, high HbA1c, high systolic blood pressure, low total protein, presence of retinopathy	–
static analysis	Wang et al. (2019) [25]	eGFR decline >5 mL/min per 1.73 m^2^/year	T2D (N = 128)	a family history of diabetes, higher degree of proteinuria, higher grades of glomerular pathology class, higher interstitial fibrosis and tubular atrophy, interstitial inflammation	–
static analysis	Fujii et al. (2023) [11]	eGFR decline ≥10 %/year	T2D, T1D (N = 10,712,577)	high systolic blood pressure, poor or strict diabetes control, increased urinary protein excretion, decreased blood hemoglobin levels	–
static analysis	Xie et al. (2023) [33]	eGFR decline >5 mL/min/1.73 m^2^/year	T2D (N = 526)	anemia	–
static analysis	Li et al. (2023) [26]	eGFR decline >5 mL/min/1.73 m^2^/year	T2D (N = 573,860)	lifestyles (skipping breakfast, regular smoking, a lack of habitual exercise, late-night dinners, non-refreshing sleep, and a high alcohol intake)	–
static analysis	Yoshida et al. (2020) [12]	eGFR decline ≥10 %/year	T1D, T2D (N = 9010)	older age, higher basal eGFR, higher urinary albumin to creatinine ratio , higher systolic blood pressure	–
ML	Inaguma et al. (2020) [20]	eGFR decline ≥30 % within 2 years	CKD patients (N = 9911)	urine protein, hemoglobin, total cholesterol, uric acid	0.73
ML	Hirakawa et al. (2022) [21]	eGFR decline ≥10 %/year	DKD patients (N = 150)	systolic blood pressure, urinary albumin, and urinary metabolites such as threnodic acid, trigonelline, urinary metabolite (urinary 1-Methylpyridin-1- ium )	0.775

The definition of RD varies somewhat across studies. Several predictors of RD, such as HbA1c, have been identified.

The aim of this study is to develop an ML tool that accurately predicts RD using input from common laboratory tests performed in diabetes care, selecting tests and their timing that contribute to the prediction. The importance and originality of this study is that it explores factors associated with RD from 1202 clinical examinations in 3438 T2D patients. Based on the performance of prior studies [20,21], our goal is to achieve an ROC-AUC above 0.775 for the task of predicting RD among diabetes patients. By selecting laboratory tests by balancing test frequency and contribution to prediction, we aim to have a model using a limited number of laboratory tests, with the expectation that such a parsimonious model is more likely to be adopted in clinical settings. Identifying patients who are likely to develop RD gives physicians early warning and allows them to refer patients to a nephrologist. The identification of the tests useful to the model may contribute to the understanding of RD.

## 2 Methods

### 2.1 Definition of RD and study design

According to the KDIGO 2012 clinical practice guidelines [5], RD is characterized by a decrease in eGFR over 1 year that is greater than 5 mL/min/1.73 m^2^/year. RD has been defined in different ways across studies (Table 1). While some studies define RD as a decline in the slope of eGFR [[23], [24], [25], [26],^33^], others use the criterion of a loss of eGFR percentage per year exceeding a certain threshold [2,[7], [8], [9], [10], [11], [12]]. In our observation, practicing physicians at the University of Tokyo Hospital generally decide that RD is indicated when eGFR is below 60 mL/min/1.73 m^2^, indicating kidney disease, and eGFR declines greater than 5 mL/min/1.73 m^2^/year, as estimated via visual examination of multiple eGFRs to estimate the slope, accounting for the effects of bias from the testing date and minor fluctuations.

Based on these studies and observations, we set this study's criteria for RD as having an eGFR below 60 mL/min/1.73 m^2^ and observing a decrease of 5 mL/min/1.73 m^2^ or more from 1 year ago to the *indication point* (the consultation date on which a patient measures eGFR and an indication of *RD* or *not_RD* is made), after smoothing using the Lowess method [34,35]. This smoothing was used to reduce the impact of different numbers of eGFR tests. As a first step, we classified all indications as RD or not_RD.

**Fig. 1:**
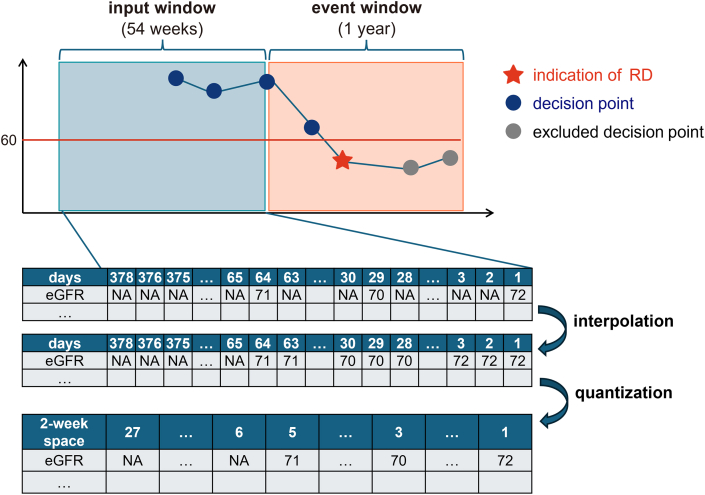
Illustration of input windows, decision points, event windows, and indication points.

Predictions are made at a *decision point* (a date on which a patient measures eGFR and an RD prediction is made), using inputs from an *input window* (a window from the decision point to 54 weeks prior) (Fig. 1). The prediction is whether RD will be indicated in the *event window* (a window of 1 year following the decision point). For each patient, we ceased predictions once RD was indicated. The timing avoids making predictions at inappropriate times - when RD has already been indicated, when there is not at least 1 eGFR measurement in the input window, and when there are no measurements in the event window. Our ML task is to predict at each decision point if the indication of RD will occur within the event window based on laboratory test values collected within the input window.

To prevent the misclassification of RD, one possible approach is to set a threshold for the number of eGFR tests or the value of eGFR like Nojima's et al. [35] However, the purpose of our ML model is designed to identify patients at high RD risk and quickly refer them to specialists. To ensure that no potential cases are overlooked, we did not set such a threshold based on the advice of nephrologists. Therefore, we classified all cases with a potential risk of RD as RD, regardless of the number of eGFR tests conducted.

Moreover, we adopted a policy of using multiple decision points from a single patient. We think that using multiple data points matches the way that physicians treat patients over a trajectory of visits and can lead to better predictive models because the scale of data available for model training is larger. This approach is a good way to build better models with limed EHR data. This method has also been shown to be effective in a previous study [20].

### 2.2 Data source

Our participant-selection process used 2 data sources, electronic health records (EHRs) at the University of Tokyo Hospital and the Japan diabetes comprehensive database project based on an Advanced electronic medical record system (J-DREAMS) [36]. J-DREAMS is a database of patients with diabetes in Japan launched by the National Center for Global Health and Medicine and the Japan Diabetes Society. J-DREAMS registers both outpatients and inpatients with diabetes who are treated by diabetologists at participating institutions. The University of Tokyo Hospital is also one of the participating institutions in the J-DREAMS project. The database includes standardized information on patients' physical conditions, medical histories, and complications. In this study, we extracted demographic and laboratory test data from EHRs at the University of Tokyo Hospital, and diagnostic codes (ICD-10) and information of kidney dialysis and kidney transplant from J-DREAMS. We obtained data from a general hospital in Japan. This hospital offers a wide range of medical services beyond just diabetes care, allowing for comprehensive consideration of patients’ overall health conditions. Compared to data from a single clinic specializing in a particular department, the data from this general hospital includes a broader spectrum of patient backgrounds and comorbidities, making it more representative of the general patient population. Therefore, we think that the data from this general hospital provides a more reliable foundation for evaluating RD and offers findings that are more applicable to a general patient population compared to data from a single clinic.

**Fig. 2:**
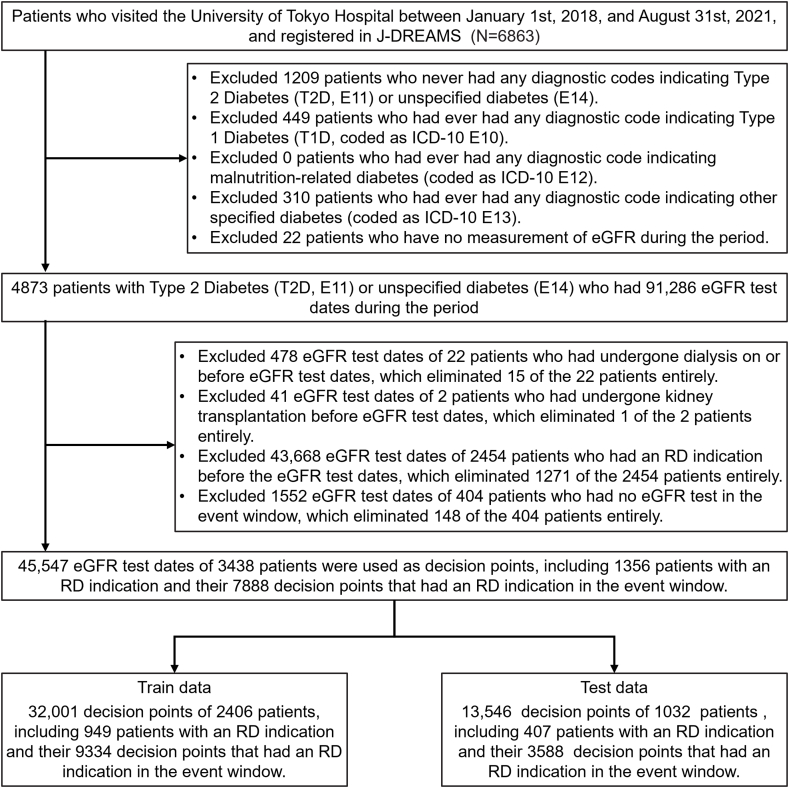
Patient selection process.

Initially, our data consisted of patients who visited the University of Tokyo Hospital between January 1st, 2018, and August 31st, 2021, and registered in J-DREAMS (Fig. 2). We excluded patients who had never had any diagnostic codes indicating T2D (coded as ICD-10 E11) or unspecified diabetes (coded as ICD-10 E14), and then excluded patients who had ever had any diagnostic code indicating Type 1 Diabetes (T1D, coded as ICD-10 E10), malnutrition-related diabetes (coded as ICD-10 E12), or other specified diabetes (coded as ICD-10 E13). We then filtered down to the patients who had at least 1 eGFR measurement during the period, as the model makes predictions at decision points defined by the occurrence of an eGFR measurement. In our analysis, each patient had multiple visits during the analysis period, so events of dialysis and kidney transplant may have changed during the analysis period. Therefore, we checked each eGFR test date and excluded test dates where patients had a kidney transplant or kidney dialysis before the test date. Patients may have had more than one instance of these excluded conditions. We also excluded eGFR test dates if they were after or on the date of an RD indication or if there were no other eGFR test dates within 1 year after the eGFR test date. Each remaining eGFR test date between January 1st, 2018, and August 31st, 2020, was used as a decision point. For each decision point, we prepared labels to indicate whether RD is included in the event window and made a set of input values that were included in the input window for that decision point.

Dialysis and kidney transplant event are not relevant to the decision point and event window decision process. These events were used only to ensure that no data were recorded after the date of dialysis and kidney transplant.

### 2.3 Laboratory tests used as features

Laboratory test values at each time were used as features because we expected that the rate of change of each test value might be significant. We obtained laboratory data from the EHR database that used standardized Japanese Laboratory Code Version 10 (JLAC10) [37]. As preliminary data processing, we used the nearest-neighbor method to interpolate the sparse data, producing daily values for each patient and each input window. We did not extrapolate (In the example shown in Fig. 1, eGFR tests were performed 1, 29, and 64 days before the decision point, and the eGFR test values from 2 to 28 days ago and from 30 to 63 days ago were then interpolated.). All cases where there was no value in the input window were treated as missing. Then, we quantized the input data into equally-spaced values (with a 2-week spacing) [38] and calculated the logarithm of the average as a representative value for the 2-week period. The test results were transformed into tabular data. The features were named, in the order from newest to oldest test, as “1st_space_of_LabTest”, “2nd_space_of_LabTest ", …, “27th_space_of_LabTest”. Thus, each laboratory test has 27 features within the 54-week window.

### 2.4 ML models

We designed an ML model that takes as input laboratory test values spanning over the past 54 weeks and outputs a score representing the probability of whether the patient will have an indication of RD within the next year and a binary decision based on whether the probability is greater than or equal to 0.5. The score ranges from 0 to 1, with a threshold of 0.5 indicating an equal probability of having an indication of RD within the next year versus not having one. We used LightGBM [29] for the model architecture. LightGBM is a highly efficient algorithm capable of handling large-scale tabular datasets, making it well-suited for our research using various kinds of laboratory tests. LightGBM also treats missingness as a feature. Previous studies of similar T2D prediction tasks showed good performance using LightGBM [31,32], so we selected this algorithm. RFECV is a feature selection algorithm eliminating features based on feature importance. By reducing the feature set, RFECV contributes to a simpler and more generalizable model. RFECV has an advantage over other feature selection methods in that it does not rely on a specific algorithm and adaptively identifies the optimal features for the model. A similar method to RFECV is a feature selection based on feature importance. RFECV aims to find the optimal subset of features, whereas feature importance ranks all features based on their contribution to the model. Since the goal of this work is to find optimal subsets of laboratory tests to indicate RD, RFECV was used. Combining RFECV with LightGBM allows for the construction of a highly accurate model while improving interpretability.

**Fig. 3:**
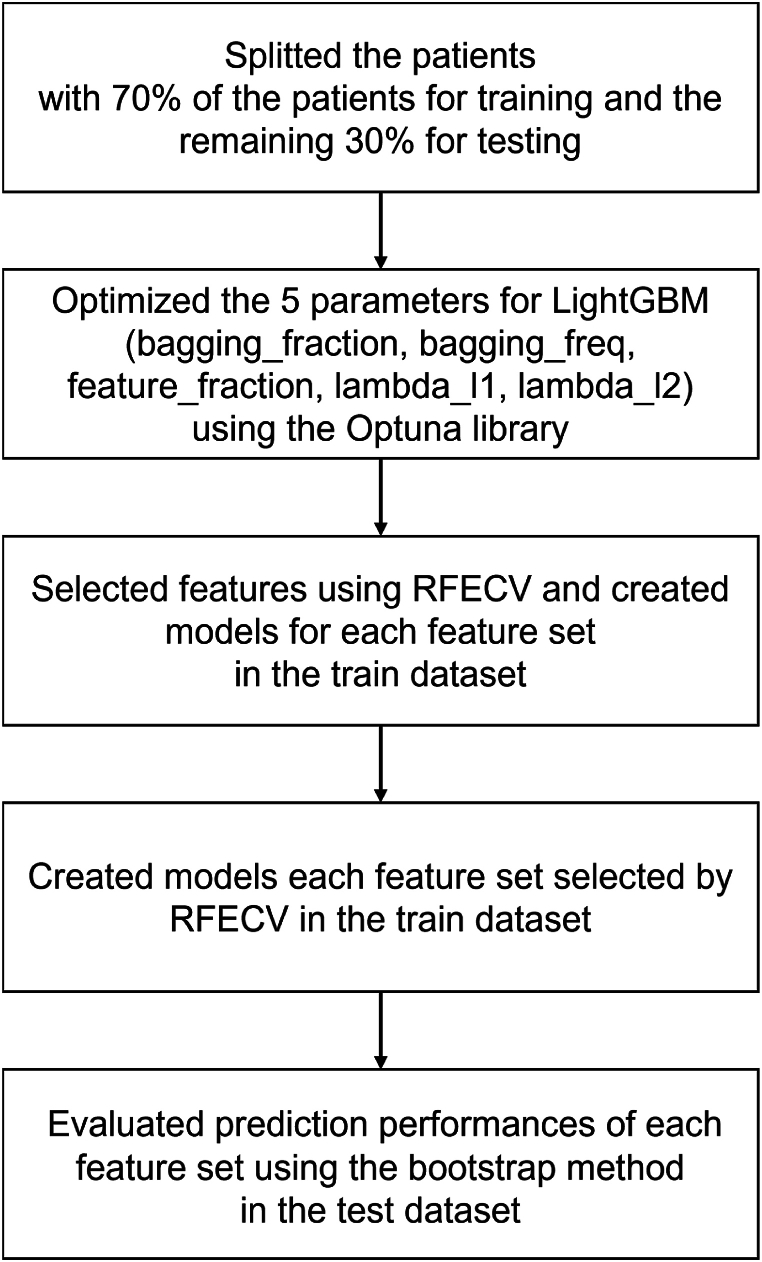
The process of feature selection and leaning models.

To prevent to over fitting and data leakage, we performed the evaluation by combining the holdout verification and the bootstrap methods (Fig. 3). First, we randomly split the patients using a 70–30 ratio, with 70 % of the patients for training and the remaining 30 % for testing. There is no overlap between the patients in the training and test datasets. Second, we optimized the 5 parameters for LightGBM (bagging_fraction, bagging_freq, feature_fraction, lambda_l1, lambda_l2) using the Optuna library [39] and the training dataset (Table 2). We searched for the best parameters using 100 trials to maximize the area under the precision-recall curve (PR-AUC) using Optuna library. Third, we selected features using RFECV and created models for each feature set selected by RFECV in the training dataset, using parameters optimized by Optuna. While applying RFECV, we eliminated 5 % of the features at each iteration while there were more than 40 features. We then eliminated features one by one, down to 1 feature. We created models for each feature set selected by RFECV using the training dataset. To prevent overfitting, we set the early stopping option in LightGBM. If no improvement in PR-AUC was observed after 50 iterations, training was stopped. Finally, to evaluate prediction performances of each feature set, we used the bootstrap method [41] in the testing dataset. We resampled from the test dataset, with replacement, 1000 times to generate 1000 samples. By calculating the predictive accuracy for each sample, we determined the 95 % CI. We confirmed overfitting by visualizing performance metric trends for both the training and test datasets. If the performance metrics of the test dataset were lower than that of the metrics of the training dataset, we can determine that overfitting did not occur.

**Table 2:** LightGBM Model details.

Configure	Value	Tune of Optuna
bagging_fraction	1	✓
bagging_freq	0	✓
boosting	Gbdt	
deterministic	TRUE	
device type	Cuda	
feature_fraction	0.852	✓
feature_pre_filter	FALSE	
force_col_wise	TRUE	
lambda_l1	9.998	✓
lambda_l2	2.561	✓
learning_rate	0.05	
max_depth	10	
metric	PR-AUC	
min_child_samples	100	
n_jobs	−1	
n_estimater	1000	
num_leaves	50	
objective	cross entropy loss	
stratified	TRUE	
threshold	0.5	
feature importance	gain	
GPU	cuda12.2	
library	Python 3.11, Scikit-lean 2.2.0 [40] and LightGBM 3.3.1 [29], Optuna 2.10.0 [39].	

Five of the parameters were optimized with Optuna.

### 2.5 Statistical methods and data usage

We analyzed the characteristics of patients in the dataset using means, standard deviation, and frequency counts. We performed all statistical analyses using custom Python code. *P* values comparing RD and not_RD were computed using the Fisher exact test for categorical variables and missing rates and Mann-Whitney *U* test for continuous variables.

We assessed the predictive accuracy of classification models using the receiver operating characteristic (ROC) area under the curve (AUC), PR-AUC, accuracy rate, and F1-score. ROC captures the relationship between the true positive rate (sensitivity) on the y-axis and the false positive rate on the x-axis. The ROC curve varies based on the selection of threshold, with a tradeoff between true positives and false positives. The AUC, calculated by numerically integrating the area under the ROC curve, assesses overall discriminatory performance across thresholds, with a higher ROC-AUC score indicating better overall discriminatory performance. Similarly, the PR curve has the proportion of correctly identified positive cases among all cases predicted as positive (precision) on the y-axis and the proportion of correctly identified positive cases among all actual positive cases (recall) on the x-axis, and the PR-AUC is the area under the precision-recall (PR) curve. A higher PR-AUC score signifies better precision in identifying positive cases while mitigating false positives [42]. The accuracy rate is the percentage of all predictions in a given dataset that are correctly predicted. The F1-score is the harmonic mean of precision and recall.

In addition, selected variance inflation factors (VIF) are calculated to identify the effects of multicollinearity. VIF is one indicator of multicollinearity, and if the value is large, it may be better to remove the variable from the analysis. In general, a VIF >10 indicates strong multicollinearity. We calculate correlation using the Pearson product-moment correlation coefficient method. The VIFs are calculated using statsmodels 0.14.2 [43].

### 2.6 Ethical considerations

This study was approved by the Institutional Review Board of the University of Tokyo School of Medicine (approval number: 10,705) and was conducted in accordance with the Declaration of Helsinki. This was a retrospective, non-interventional database study without patient involvement. Confidentiality was safeguarded by the University of Tokyo Hospital. According to the Guidelines for Epidemiological Studies of the Ministry of Health, Labour and Welfare of Japan [44], written informed consent was not required. Information about the current study was available to patients on a website, and patients have the right to cease registration of their data at any time [45].

## 3 Results

### 3.1 Patient characteristics

**Fig. 4:**
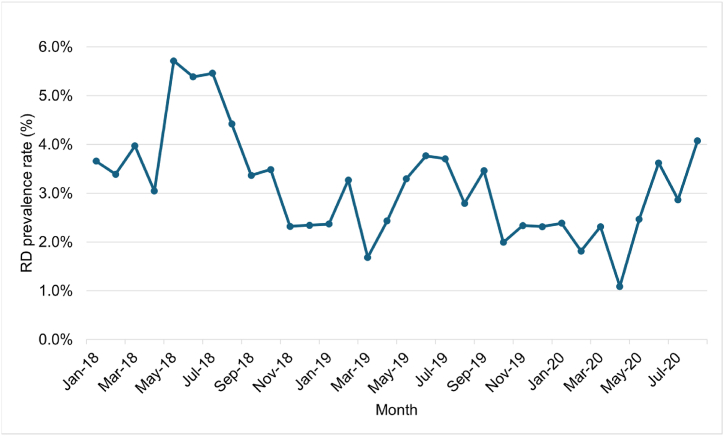
Prevalence rate of RD.

Our subject selection process (Fig. 2) started with 6863 candidates and yielded 3438 patients and their 45,547 decision points, including 1356 patients with an RD indication and their 7888 decision points that had an RD indication in the event window. We used the data from 2406 patients (70 %) for training and the data from 1032 patients (30 %) for testing. Our study design allowed for each patient to have multiple records. When tallied per patient, there was an average of 13.2 decision points per patient, with a standard deviation of 10.9. There was a possibility of overfitting some patients, but we addressed this using the measures described in the “ML models” section. The prevalence of RD varied by month (Fig. 4), with an average prevalence of 3.1 %. The monthly prevalence of RD was approximately 3 % throughout the observation period. With preliminary data processing, 27 features were generated for each of 1202 types of laboratory test, resulting in a total of 32,454 features before RFECV. The detail of 1202 types of laboratory test shows in appendix Table 1.

### 3.2 Performance metrics in training dataset and testing dataset

**Fig. 5:**
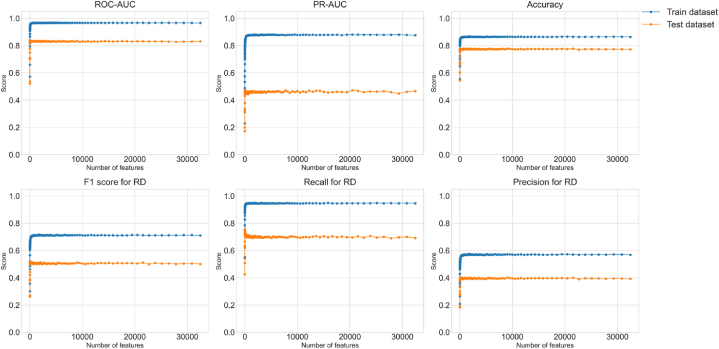
6 performance metrics of the training and test datasets.

To check for overfitting, we examined the 6 performance metrics (ROC-AUC, PR-AUC, accuracy, recall, precision, F1-score) of the training and test datasets (Fig. 5). We used PR-AUC in the early stopping setting (Fig. 5). The results for all 6 performance metrics show that the predictive accuracy of models with a certain number of features or more was roughly the same, we achieved the target predictive accuracy, and there was no sign of higher accuracy under specific feature set conditions in either training or testing, indicating no overfitting.

### 3.3 Prediction performance for various numbers of feature

**Fig. 6:**
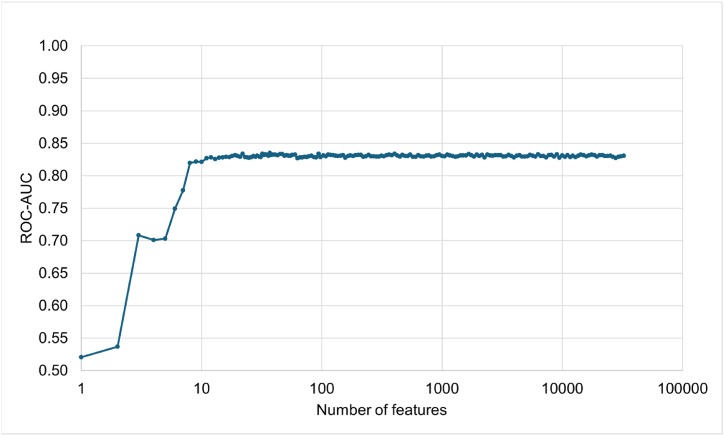
Prediction performance for various numbers of features.

We assessed using different numbers of features for the prediction, varying from 32,454 features down to 1 feature (Fig. 6). Using all 32,454 features containing 1202 types of laboratory tests produced an average (95 % Cl) ROC-AUC of 0.831 (0.822, 0.839). The ROC-AUC remained almost unchanged when the number of features reduced from 32,454 to 8 and kept good performance above our goal of 0.775 until 6 features (Table 3). We chose the 8-feature version as our model because the ROC-AUC was high with 8 features but dropped sharply with 7 features. The 8-feature model achieved average (95 % Cl) ROC-AUC, PR-AUC, Accuracy, and F1-scores of 0.820 (0.811, 0.829), 0.430 (0.410, 0.451), 0.754 (0.747, 0.761), and 0.500 (0.485, 0.515), respectively. The 8 remaining features were the 3rd space of mean corpuscular hemoglobin (MCH), 1st space of γ-glutamyl trans peptidase (γ-GTP), 4th space of creatinine (Cre), 4th space of HbA1c, 3rd space of high density lipoprotein cholesterol (HDL-C), 11th space of eGFR, 1st space of hematocrit (Hct), and 1st space of eGFR. The laboratory test characteristics for the 3438 patients shows that decision points of RD had a higher MCH, higher γ-GTP, higher Cre, lower HbA1c, lower HDL-C, lower eGFR, and lower Hct than those of not_RD (Table 4). Furthermore, decision points of RD had more missing values for MCH, Cre, HbA1c, HDL-C, and eGFR than those of not_RD.

**Table 3:** Mean (95 % CI) prediction performance from 1 to 8 features and 32,454 features.

Number of features	Number of types of laboratory tests	Feature added from a model with one less feature	ROC-AUC	PR-AUC	Accuracy	F1-Score
1	1	3rd space of MCH	0.521 (0.508, 0.533)	0.172 (0.163, 0.181)	0.544 (0.536, 0.553)	0.268 (0.256, 0.280)
2	2	1st space of γ-GTP	0.537 (0.522, 0.550)	0.199 (0.187, 0.213)	0.603 (0.594, 0.611)	0.261 (0.246, 0.275)
3	3	4th space of Cre	0.708 (0.697, 0.720)	0.321 (0.303, 0.341)	0.667 (0.659, 0.676)	0.383 (0.369, 0.398)
4	4	4th space of HbA1c	0.701 (0.690, 0.712)	0.321 (0.304, 0.338)	0.676 (0.668, 0.683)	0.387 (0.372, 0.401)
5	5	3rd space of HDL-C	0.703 (0.693, 0.715)	0.312 (0.295, 0.330)	0.662 (0.654, 0.670)	0.382 (0.369, 0.396)
6	6	11th space of eGFR	0.749 (0.739, 0.760)	0.337 (0.318, 0.354)	0.680 (0.673, 0.688)	0.421 (0.407, 0.435)
7	7	1st space of Hct	0.777 (0.768, 0.786)	0.379 (0.360, 0.400)	0.723 (0.716, 0.730)	0.443 (0.427, 0.456)
8	7	1st space of eGFR	0.820 (0.811, 0.829)	0.430 (0.410, 0.451)	0.754 (0.747, 0.761)	0.500 (0.485, 0.515)
32,454	1202	(all features)	0.831 (0.822, 0.839)	0.466 (0.445, 0.489)	0.772 (0.765, 0.779)	0.500 (0.484, 0.516)

The ROC-AUC remained almost unchanged when the number of features reduced from 32,454 to 8 and kept high performance above our goal of 0.775 until 6 features.

**Table 4:** Laboratory test characteristics for the 8 selected features.

Characteristic/Laboratory test	RD (N = 7888)	not_RD (N = 37,659)	*P* value
**Sex**			<0.001
Male, n (%)	5156 (65.4)	23,745 (63.1)	
Female, n (%)	2732 (34.6)	13,914 (36.9)	
Age	69.3 (10.7)	65.0 (12.2)	<0.001
**3rd space of MCH, mean (SD)**	30.4 (2.5)	30.4 (2.2)	0.003
Missing	963 (12.2)	3902 (10.4)	<0.001
<27.5, n (%)	658 (8.3)	2842 (7.5)	
27.5–33.2, n (%)	5661 (71.8)	28,887 (76.7)	
≥33.2, n (%)	606 (7.7)	2028 (5.4)	
**1st space of γ-GTP, mean (SD)**	61.1 (109.4)	44.4 (63.5)	<0.001
Missing	695 (8.8)	3186 (8.5)	0.31
<50, n (%)	5149 (65.3)	26,407 (70.1)	
50–100, n (%)	1134 (14.4)	5354 (14.2)	
≥100, n (%)	910 (11.5)	2712 (7.2)	
**4th space of Cre**			
Male, mean (SD)	1.1 (0.6)	1.1 (1.4)	<0.001
Missing	720 (9.1)	2355 (6.3)	<0.001
<0.65	197 (3.8)	3375 (14.2)	
0.65–1.09	3192 (61.9)	15,688 (66.1)	
≥1.09	1047 (20.3)	2327 (9.8)	
Female, mean (SD)	0.8 (0.5)	0.7 (0.9)	<0.001
Missing	359 (4.6)	1427 (3.8)	<0.001
<0.46	93 (3.4)	1216 (8.7)	
0.46–0.82	1756 (64.3)	10,356 (74.4)	
≥0.82	524 (19.2)	915 (6.6)	
**4th space of HbA1c, mean (SD)**	7.0 (1.1)	7.2 (1.1)	<0.001
Missing	1929 (24.5)	7099 (18.9)	<0.001
<6	867 (11.0)	2821 (7.5)	
6-7	2202 (27.9)	12,010 (31.9)	
7-8	1967 (24.9)	10,905 (29.0)	
≥8	923 (11.7)	4824 (12.8)	
**3rd space of HDL-C, mean (SD)**	57.6 (18.0)	59.8 (17.5)	<0.001
Missing	2864 (36.3)	9301 (24.7)	<0.001
hdlc<40	703 (8.9)	2529 (6.7)	
0-120	4287 (54.3)	25,660 (68.1)	
≥120	34 (0.4)	169 (0.4)	
**11th space of eGFR, mean (SD)**	64.4 (21.0)	78.4 (25.9)	<0.001
Missing	2611 (33.1)	9151 (24.3)	<0.001
<15	111 (1.4)	786 (2.1)	
15-30	154 (2.0)	401 (1.1)	
30-44	489 (6.2)	855 (2.3)	
45-59	972 (12.3)	1386 (3.7)	
60-89	3195 (40.5)	18,493 (49.1)	
≥90	356 (4.5)	6587 (17.5)	
**1st space of Hct**			
Male, mean (SD)	39.2 (6.3)	41.9 (5.5)	<0.001
Missing	61 (0.8)	609 (1.6)	<0.001
<37.1	1730 (33.6)	3955 (16.7)	
37.1–50.7	3287 (63.8)	18,437 (77.6)	
≥50.7	78 (1.5)	744 (3.1)	
Female, mean (SD)	37.8 (4.9)	39.9 (4.3)	<0.001
Missing	48 (0.6)	310 (0.8)	0.13
<34.0	584 (21.4)	1257 (9.0)	
34.0–44.8	1930 (70.6)	10,766 (77.4)	
≥44.8	170 (6.2)	1581 (11.4)	
**1st space of eGFR, mean (SD)**	62.5 (20.5)	77.9 (26.9)	<0.001
Missing	0 (0.0)	0 (0.0)	>0.99
<15	201 (2.5)	1096 (2.9)	
15-30	264 (3.3)	582 (1.5)	
30-44	799 (10.1)	1185 (3.1)	
45-59	1639 (20.8)	2111 (5.6)	
60-89	4502 (57.1)	24,085 (64.0)	
≥90	483 (6.1)	8600 (22.8)	

The features were named, in the order from newest to oldest test with a 2-week spacing, as “1st_space_of_LabTest”, “2nd_space_of_LabTest “, …, “27th_space_of_LabTest”.

### 3.4 Features statistical characteristics

We calculated VIF to check for multicollinearity. The VIF values of the selected 8 feature (Table 5) were all well below 10, showing a lack of strong multicollinearity.

**Table 5:** VIF in 8 features.

Feature	VIF
3rd space of MCH	2.857
1st space of γ-GTP	1.022
4th space of Cre	4.281
4th space of HbA1c	1.860
3rd space of HDL-C	1.483
11th space of eGFR	1.490
1st space of Hct	1.304
1st space of eGFR	3.427

## 4 Discussion

### 4.1 Prevalence rate

The monthly prevalence of RD in our study was 3.1 %, translating to 31.8 % annually (1 - (1–0.031)ˆ12). Yokoyama et al. [10], Fuji et al. [11], Yoshida et al. [12], Hirakawa et al. [21], and Nojima et al. [35] reported prevalence rates of 10 %, 11 %, 12 %, 21 %, and 20 % in diabetes patients, respectively. This is higher than seen in previous studies. We think that these differences in prevalence rates among the previous studies are due to differences in the definition of RD. The most similar study is by Nojima et al. because we used the same smoothing method (Lowess method) to determine RD. Our prevalence rate was 10 % lower than Nojima's prevalence rate. We confirmed the details of Nojima's method: their study is a 9-year follow-up study, and they excluded patients with an eGFR of 60 mL/min/1.73 m^2^ or less in the first 2 years. Therefore, we consider that our higher prevalence rate is due to the exclusion of severe patients in the previous studies. Moreover, in the previous study targeting Japanese individuals in a medical checkup system (“Tokuteikenshin” program) [11], the average eGFR value (standard deviation, SD) for the RD group was 79.58 (20.92), whereas in our study, the average eGFR value (SD) for the RD group was 62.5 (20.5). Compared to the previous studies [11], the eGFR values in our study were approximately 17 mL/min/1.73 m^2^ lower, indicating a tendency towards greater severity in patients. Furthermore, the fact that the University of Tokyo Hospital is a Clinical Research Core Hospital may also have an impact, as it likely attracts patients with relatively severe conditions. Our determination of RD is based on 45,547 eGFR tests from 3438 patients. As the University of Tokyo Hospital is a Clinical Research Core Hospital, it gathers a diverse patient population and conducts extensive laboratory tests. The extensive data collected provides a robust basis for our conclusions.

### 4.2 Evaluation of the predictive accuracy

The average (95 % CI) ROC-AUC of the 8-feature model was 0.820 (0.811, 0.829). While this appears to be a useful level of performance in the context of ML, it is crucial to consider the potential clinical implications of false positives and false negatives. A false positive case could lead to unnecessary medical treatment and laboratory tests for patients, resulting in a decline in their quality of life and adding a burden to human resources and finances in the healthcare system. Conversely, a false negative could result in delayed diagnosis and treatment for patients truly in need such as specialized medical care. A previous study [21] had a slightly different task design but had similar but slightly worse results with an ROC-AUC of 0.775. The 8-feature model also has a fairly good average (95 % CI) accuracy of 0.754 (0.747, 0.761), better than the 0.687 accuracy reported in a previous study [21]. Although the risk of false positives and negatives must be acknowledged, our model provides more reliable predictive performance than the previous study [21]. The mechanism of RD progression is still largely unclear, and established treatments are not currently available. Therefore, it is difficult to evaluate the utility of any level of predictive accuracy. Once effective treatments are developed, though, our model's level of performance may be useful in 3 key areas: healthcare economics, patient quality of life, and healthcare resources. Our ML model may make it possible for the health care system to provide additional RD-related medical care (with its associated expense) to patients with a high probability of RD and eliminate unnecessary RD-related medical care (and expense) on patients with a low probability of RD. Our ML model's accurate predictions may contribute to maintaining the quality of life of patients, helping patients in need, and avoiding unneeded treatments for those not facing likely RD. Accurate predictions enable efficient use of healthcare resources, including an appropriate division of roles between primary care physicians and specialists, with the limited number of nephrologists being assigned to treat patients who are likely to have RD and require more specialized care. Conversely, using an ML model with low performance may lead to inaccurate predictions and increased unnecessary medical procedures, potentially resulting in higher healthcare costs. For nephrologists, inappropriate early referral of patients less likely to have RD could be disadvantageous, leading to increased congestion and workload in hospitals, and inefficient use of healthcare resources. The results of the model may help minimize healthcare costs, minimize patient burden, and optimize the use of healthcare resources.

The average (95 % CI) of the PR-AUC and the F1-score were reasonable, at 0.430 (0.410, 0.451) and 0.500 (0.485, 0.515), respectively. Since previous studies did not evaluate PR-AUC or F1-score, a direct comparison of performance cannot be made. Our RD prediction was a classification problem with imbalanced classes, where decision points of RD accounted for 17.3 % (7888/45,547) of all decision points. In cases with imbalanced classes, PR-AUC and F1-score tend to provide a stricter evaluation, often resulting in lower values, by highlighting the model's ability to detect the minority class. Now that we have achieved the target value for ROC-AUC, we would like to consider ways to increase the PR-AUC and F1-score. We considered applying methods to handle imbalanced distribution, such as down sampling or up sampling methods, to address the imbalanced distribution and improve the PR-AUC and F1-score. However, these strategies tend to fit to the smaller class, which increases the risk of overfitting. There is a trade-off between the improvement of the imbalance distribution and the risk of overfitting. Therefore, we did not apply a method for handling unbalanced data.

### 4.3 Evaluation of the selected laboratory tests

There was a small difference in ROC-AUC between using all 32,454 features containing 1202 types of laboratory tests and using just 8 features containing 7 types of laboratory tests. This implies that the 7 laboratory test types (MCH, γ-GTP, Cre, HbA1c, HDL-C, eGFR, and Hct) are essential to achieving high ROC-AUC performance.

Measurements of eGFR and Cre can indicate decreased renal function [5], and these laboratory tests have been reported as associated factors for RD in previous studies [4,[8], [9], [10],^12^,^13^].

Except for the 11th space of eGFR, the selected features were measured close to the decision point. The result of use of eGFR values from different time points indicates the importance of capturing both current status and recent historical context. The 1st and 11th spaces of eGFR represent 2 samples within the past 5 months, and their values were lower in decision points with RD than in those with not_RD. Since eGFR was the most frequently selected feature, trends in eGFR appear to be highly important to prediction.

HbA1c was reported in a previous study as an associated factor for RD [10]. Poor glycemic control and HbA1c variability are associated with RD [46,47].

A high HbA1c and low HDL-C were reported as an associated factor for DKD [48,49]. A 12 % increased risk of DKD onset per 1 % increase in HbA1c. For HDL, a 6 % decreased risk of DKD onset per 10 mg/dL increase in HDL was reported [48].

The importance of the other 3 tests (γ-GTP, MCH, and Hct) has not been featured in prior work to our knowledge. Our results suggest that the timing of changes in these laboratory values plays a crucial role in predicting the occurrence of RD.

A high γ-GTP indicates the condition of liver function. When the liver functions poorly, the flow of blood throughout the body becomes poor, and the flow of blood to the kidneys also becomes poor. Although acute kidney injury (AKI) is a kidney-related condition distinct from RD, studies [50] have identified γ-GTP as predictive markers of AKI.

A high MCH and low Hct indicate anemia. Erythropoietin is one of the hormones which is secreted by erythropoietin-producing cells and plays a role of stimulating production of red blood cells. When kidney function impairs due to various reasons including diabetic mellitus or hypertension, erythropoietin cannot be made, leading to anemia [33].

A recent study reported that anemia is an independent risk factor for RD in T2D [33].

These laboratory tests are well-known to be somewhat associated with the kidneys. Our results suggest that predicting RD requires the consideration of multifaceted measurements related to renal function.

It should be noted that it cannot be said that the test values not selected by RFECV

are unrelated to renal function. First, some test values may not have enough data for RD modeling, and may have been overlooked in this experiment even if they were related to RD. Second, test values may not have been selected because they happened to be correlated with the 8 selected features. These ideas could be explored by performing the same experiment with a different dataset.

## 5 Limitations

Our study has notable limitations. First, the data were sourced from a single hospital, limiting generalizability. Further research using diverse hospital data is needed. Secondly, missing values in the data source may influence the importance of features. The proposed model treats missing values as a categorical feature, resulting in outcomes that include not only the magnitude of lab test values but also the presence or absence of lab tests. Thirdly,

due to the limited understanding of the mechanism of RD, it was challenging to accurately identify and control potential confounding factors among laboratory tests. As a result, we did not explicitly adjust for confounding variables in our analysis. These results therefore need to be interpreted with caution. Fourthly, the features selected by RFECV may not necessarily be the optimal combination. Although RFECV improves the quality of selection by combining RFECV with cross-validation, there remains a slight possibility that it will converge to locally optimal features. Fifthly, including the period of the COVID-19 pandemic in the data source may contain trends different from the current treatment state. Sixthly, there is a tendency for more frequent eGFR tests among severe patients, potentially leading to biased results. Finally, the number of test values varies from patient to patient, which may lead to different accuracy of predictions for different patients. In particular, features from patients with only a small number of laboratory tests are interpolated using nearest neighbor methods, resulting in reduced resolution of the timing of examinations.

## 6 Conclusions

The proposed model accurately predicts RD in DKD patients, aiding physicians in understanding RD. The contributing laboratory tests (MCH, γ-GTP, Cre, HbA1c, HDL-C, eGFR, and Hct) may serve as potential alternative biomarkers for RD. In this experiment, we conducted experiments using EHRs from a hospital with a relatively high number of severe patients. We think that this technology can be applied not only to identify RD patients early and start treatment, but also to improve the efficiency of subject selection for clinical trials. Going forward, we intend to expand our study to include EHRs from multiple facilities and further strengthen the evidence base.

## CRediT authorship contribution statement

**Eri Nakahara:** Writing – original draft, Visualization, Software, Methodology, Conceptualization. **Kayo Waki:** Writing – review & editing, Supervision, Funding acquisition, Data curation, Conceptualization. **Hisashi Kurasawa:** Writing – review & editing, Software, Methodology. **Imari Mimura:** Writing – review & editing, Supervision. **Tomohisa Seki:** Supervision, Data curation. **Akinori Fujino:** Supervision, Methodology. **Nagisa Shiomi:** Supervision. **Masaomi Nangaku:** Writing – review & editing, Supervision. **Kazuhiko Ohe:** Supervision.

## Data availability

The data in this study are not openly available because of restrictions imposed by the research ethics committees that approved this study.

## Code availability

The underlying code for this study is not publicly available but may be made available to qualified researchers on reasonable request from the corresponding author.

## Declaration of competing interest

The authors declare the following financial interests/personal relationships which may be considered as potential competing interests: Eri Nakahara, Hisashi Kurasawa, Imari Mimura, Tomohisa Seki, Akinori Fujino, Nagisa Shiomi, Masaomi Nangaku, Kazuhiko Ohe reports financial support was provided by 10.13039/501100004721The University of Tokyo and Nippon Telegraph and Telephone Corporation (NTT) in a joint research program at the 10.13039/501100004721University of Tokyo Center of Innovation, Sustainable Life Care, and the Ageless Society dedicated to Self-managing Healthcare in the Aging Society of Japan.

Kayo Waki reports financial support was provided by 10.13039/501100004721The University of Tokyo and 10.13039/100010160NTT in a joint research program at the 10.13039/501100004721University of Tokyo Center of Innovation, Sustainable Life Care, and the Ageless Society dedicated to Self-managing Healthcare in the Aging Society of Japan. Eri Nakahara, Hisashi Kurasawa, Akinori Fujino, Nagisa Shiomi reports a relationship with NTT, Tokyo, Japan that includes: employment. Eri Nakahara, Hisashi Kurasawa, Imari Mimura, Tomohisa Seki, Akinori Fujino, Nagisa Shiomi, Masaomi Nangaku, Kazuhiko Ohe reports a relationship with 10.13039/501100004721The University of Tokyo Center of Innovation (10.13039/501100009033COI) that includes: funding grants. Kayo Waki reports a relationship with 10.13039/501100004721The University of Tokyo 10.13039/501100009033COI that includes: funding grants. If there are other authors, they declare that they have no known competing financial interests or personal relationships that could have appeared to influence the work reported in this paper.

## Abbreviations

DKD: diabetic kidney disease
T2D: type 2 diabetes
RD: rapid decline
RFECV: recursive feature elimination with cross-validation
ROC-AUC: area under the receiver operating characteristic curve
ML: machine learning
eGFR: glomerular filtration rate
TNFR: TNF receptors
HbA1c: hemoglobin A1c
LDL-C: low density lipoprotein cholesterol
U-NIT: urine nitrous acid
U-UBG: urine urobilinogen
LDL-C: low density lipoprotein cholesterol
LightGBM: Light gradient boosting machine
EHRs: electronic health records
J-DREAMS: Japan diabetes comprehensive database project based on an advanced electronic medical record system
VIF: variance inflation factors
T1D: type 1 diabetes
JLAC10: Japanese laboratory code version 10
PR-AUC: area under the precision-recall curve
CI: 95 % confidence intervals
MCH: mean corpuscular hemoglobin
γ-GTP: γ-glutamyl trans peptidase
Cre: creatinine
HDL-C: high density lipoprotein cholesterol
Hct: hematocrit
SD: standard deviation
AKI: acute kidney injury
